# Secondary Compounds in Milkweed Nectar Negatively Impact Thermal Tolerance in Bumble Bees

**DOI:** 10.1002/ece3.72420

**Published:** 2025-11-09

**Authors:** Rachael Shippee, Cody Feuerborn, Allie Bradley, Hannah Jahnke, Heather M. Hines

**Affiliations:** ^1^ Department of Biology Pennsylvania State University University Park Pennsylvania USA; ^2^ Department of Entomology Pennsylvania State University University Park Pennsylvania USA

**Keywords:** *Bombus*, climate, conservation, nutrition, pollinator, toxin

## Abstract

Bumble bees play a critical role in ecosystem health and pollination, but recently, populations have been in decline worldwide. One way to improve outcomes is to increase floral resource availability, which improves nutrition while alleviating the effects of other stressors, such as climate. In addition to macronutrients, floral nectar can contain both beneficial as well as toxic secondary compounds, including cardenolides and alkaloids, that may impact these bees. Given that pesticides can affect heat shock pathways and thus impact heat tolerance, natural toxic compounds in nectar could also impact heat response. In this study, we investigate how the consumption of toxic secondary compounds found in nectar impacts a bee's ability to thermoregulate during heat stress events. We studied thermal tolerance in bumble bees fed a variety of concentrations of ouabain, a commercially available cardenolide (a class of compounds common in milkweeds (*Asclepias* spp.)) as well as a variety of honeys and milkweed nectar. We found that increasing concentrations of ouabain resulted in a diminished ability to thermoregulate during heat stress events. Ouabain had a significant impact on thermal tolerance at concentrations both at and below both field‐realistic doses and levels that impair bee behavior, with effects matched by milkweed nectar. This effect was found both in a species that tends to avoid milkweeds ( *B. impatiens* ) and a species that commonly visits milkweeds ( *B. griseocollis* ). Nevertheless, with the exception of milkweed honey, multiple honey sources showed no marked effect on heat tolerance in these bees, suggesting most floral diets may not impact thermal response. These results reveal that plant defense toxins consumed in the diet may have sublethal impacts on stress responses in these bees.

## Graphical abstract

This study shows that exposure to cardenolide toxins in floral nectar impacts heat tolerance in bumble bees at field‐realistic doses. Various honeys, aside from milkweed honey, did not impact heat tolerance in these bees. These data suggest that compounds in their diet may impact resilience of bees to global warming and that milkweed‐specialists may be less heat tolerant.

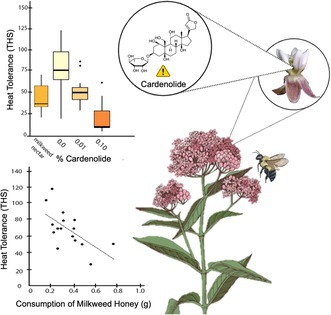

## 1 Introduction

Bumble bees are key pollinators across temperate communities and play an important role in ecosystem health (Goulson 2003). Recently, bumble bee populations have been in decline because of interacting stressors, including climate change, habitat loss, pathogens, and pesticides (Goulson et al. 2008; Soroye et al. 2020). Climate is a leading correlate with recent declines, with rising temperatures linked to population losses and range contraction (e.g., Kerr et al. 2015; Soroye et al. 2020; Martínez‐López et al. 2021; Ghisbain et al. 2023; Janousek et al. 2023). While climate change is difficult to remedy for these bees, solutions for alleviating its effects may lie in understanding the interactions between thermal tolerance and more manageable features of bumble bee landscapes, such as nutritional availability and habitat conservation.

Assays of individual bumble bees find considerable variation in heat tolerance within species (Martinet et al. 2020). Several biological factors in bumble bees have since been found to impact heat tolerance, including species (Martinet et al. 2020; Feuerborn et al. 2023), caste or sex, developmental age, and reproductive state (Feuerborn et al. 2023). Data comparing laboratory and wild bees (Feuerborn et al. 2023) and assessment of impacts of starvation (Quinlan et al. 2023) suggest that nutrition impacts heat tolerance. The effects of heat stress on colony development, for example, were less severe when colonies received optimal pollen diets (Vanderplanck et al. 2019). There is a need to better understand the aspects of diet that impact thermal resilience in these bees.

A less studied nutritional component of bumble bees' diets is the plethora of secondary compounds in their nectar (Adler 2000; Pacini et al. 2003; Egan et al. 2016; Stevenson et al. 2016; Stevenson 2020; Barberis et al. 2023). In addition to sugars, nectar contains different combinations of amino acids, vitamins and minerals, and floral volatiles, but also toxic compounds of many different types, including cardenolides and alkaloids (Adler and Irwin 2005; Manson et al. 2013). Toxic compounds may be produced in nectar as a byproduct of compounds in plant tissues that act as a deterrent against herbivores (Adler and Irwin 2005; Stevenson et al. 2016) but may also be advantageous in promoting visitation only by tolerant specialists, thus increasing pollinator fidelity without overconsumption of plant resources. For example, *Aconitum* (monkshood) nectar contains alkaloids, which promote a more specialized interaction with certain bumble bee species (Gosselin et al. 2013; Barlow et al. 2017). Most bumble bees, however, are floral generalists and thus are likely inadvertently exposed to a diversity of toxins as they sample the environment (Fontaine et al. 2006). Consumption of some of these toxic secondary compounds may be helpful to bees in low doses: natural pesticides like caffeine in small amounts can be stimulatory for bumble bee (Arnold et al. 2021) and honey bee foraging (Marchi et al. 2021) and small amounts of nicotine (Baracchi et al. 2015) and alkaloids can temporarily decrease parasitic and pathogen infection (Manson et al. 2010; Richardson et al. 2015).

Plants in Apocynaceae (dogbanes and milkweeds (*Asclepias* spp.)), as well as several other plant families, contain toxic cardenolides in vegetative tissue to deter herbivores. Cardenolides are cardiac glycosides that interfere with sodium and potassium pumps and ion balance in invertebrates, thus impairing heart and neural function (Dobler et al. 2011; Agrawal et al. 2012; Petschenka et al. 2018). Milkweed plants are vital for pollinator conservation, serving as the primary host for monarch butterfly caterpillars and a nectar source for other pollinators, which is why they are a central focus of national planting initiatives aimed at restoring habitats and supporting biodiversity (Baker and Potter 2018). Insects like monarchs that specialize in milkweeds have developed various forms of resistance to these cardenolides (Malcolm 1994; Agrawal et al. 2021).

The cardenolides produced in the leaves and stems of milkweed plants can also be found in floral nectar at lower concentrations where they can be ingested by pollinator visitors (Manson et al. 2010), which are predominantly bumble bees (Villalona et al. 2020). Anecdotal evidence supports bee intoxication and mortality after consuming these nectars in field settings, and learned avoidance of some bumble bee species (e.g., *B. impatiens* ) of milkweed has been observed in field and laboratory assays (Agrawal et al. 2012; Villalona et al. 2020; Jones et al. 2023). As such, cardenolides in nectar lead to visitation biases, as *Bombus griseocollis* in North America is a disproportionate visitor to this plant and is more tolerant of its cardenolide toxins (Villalona et al. 2020). Aside from assessing the lethal effects of high doses, the effects of cardenolide exposure on bumble bee health have not been adequately studied.

Secondary compounds like cardenolides could interplay with thermal tolerance. In honey bees, neonicotinoid pesticides affect gene expression related to heat stress responses (Manzi et al. 2020): the expression of heat shock proteins declined with high pesticide exposure (Koo et al. 2015), but smaller doses may contribute to a priming response through inducing heat shock genes, enabling improved thermoregulation during heat stress events that reduce mortality (Gonzalez et al. 2022). While cardenolides have a different mode of action, it is possible that the impaired cardiac and neural function caused by cardenolide exposure may similarly impact thermal tolerance. Cardenolides can make bees sick when exposed to high levels (Villalona et al. 2020), but there could also be effects at low levels caused by interference with the mode of action with heat resistance and stress pathways.

The purpose of this study is to further investigate the impacts of secondary compounds in nectar on heat tolerance in bumble bees, focusing on the role of cardenolide toxins. We first sought to determine whether the consumption of cardenolides at various concentrations impacts heat tolerance, as measured by the amount of time it takes to reach heat stupor at a stressful temperature. We related thermal response at these concentrations to behavioral signs of illness in bumble bees. These assessments were performed on two bumble bee species, one ( *B. impatiens* ), that is deterred by milkweed and one ( *B. griseocollis* ) that is attracted to it, using doses that straddled levels encountered in a field setting. Finally, to assess the value of these data in field settings, we tested whether different field nectar sources (honeys) that should harbor varied types of secondary compounds lead to variance in bumble bee heat tolerance.

## 2 Methods

### 2.1 Bee Sources

Experiments on *Bombus impatiens* were run on workers from commercial colonies (*n* = 8) purchased from Plant Products, (Canton, MI) and three *B. impatiens* colonies reared from wild‐caught queens from State College, PA, USA. *Bombus griseocollis* workers were sourced from two colonies reared from wild‐caught queens (State College, PA). All lab‐reared colonies were kept at 28°C and 65% humidity and fed with pollen patties made from a mix of honey bee pollens from spring flowers (Swarmbustin' Honey, Westgrove, PA, USA) and a lab‐mixed sugar solution (50% sucrose supplemented with a 0.45% amino acid booster (Amino‐B, Honey‐B‐Healthy Inc., Cumberland, MD, USA)).

### 2.2 Treatment Groups

All the below treatment groups were fed to bees prior to heat tolerance experiments (feeding procedure outlined in the next section). All treatment solutions were stored at −20°C prior to use. Treatments are further outlined in Table 1.

**TABLE 1:** Compilation of all experiments performed, and what factors were being evaluated.

Experiment	Treatments	Concentrations/type	Sugar content	Species	# bees/group
Ouabain: *B. impatiens*	Ouabain	0.0%, 0.000001%, 0.00001%, 0.00005%, 0.0001%, 0.001%, 0.01%, 0.05%, 0.10%	50%	*B. impatiens*	16–17
Milkweed sap	Milkweed sap	0%, 2%, 10%	50%	*B. impatiens*	5
Milkweed nectar	Milkweed nectar, ouabain, honey	0%, 0.00001%, 0.0001%, 0.01%, buckwheat, fireweed	20%	*B. impatiens*	12
Natural nectars	Honey	Spring blossom, buckwheat, knotweed, orange blossom, common milkweed	50%	*B. impatiens*	16–17
Ouabain: *B. griseocollis*	Ouabain	0%, 00.01%, 0.10%	50%	*B. impatiens* , *B. griseocollis*	12

#### 2.2.1 Ouabain

To test the impact of different cardenolide concentrations on bumble bee heat tolerance, nine different concentrations of the purchased cardenolide ouabain (#PHR1945 sourced from Sigma‐Aldrich) were tested on 16–17 *B. impatiens* workers per treatment, including 0.0%, 0.000001%, 0.00001%, 0.00005%, 0.0001%, 0.001%, 0.01%, 0.05%, 0.10% (weight to volume, g/mL; 0.10% = 1000 ng/uL). Ouabain is used as a proxy for field‐cardenolides, which are a mix of cardenolides (Keeler and Tu 1983), most of which are not commercially available. For these mixtures, distilled water was warmed and mixed with sucrose (granulated white pure cane sugar) to obtain a 50% sugar content, confirmed with a refractometer, and solutions were made using a serial dilution of ouabain. Treatment concentrations were chosen to straddle levels that include the natural concentration found in milkweed nectar (~0.0015% average across milkweed species) (Manson et al. 2012) (common milkweed found to be 0.00014%; Villalona et al. 2020), with upper levels representing levels that are known to make them sick (Villalona et al. 2020), and very dilute levels tested to assess potential beneficial effects at low doses.

#### 2.2.2 Milkweed Sap

To assess whether this cardenolide reflects the toxicity of natural milkweed toxins, which are known to contain a diversity of cardenolides that are not commercially available, we also ran tests on the effects of milkweed sap, where cardenolides are known to be concentrated (Nelson et al. 1981), on 5 *B. impatiens* workers per treatment. Milkweed sap was collected into a 50 mL plastic container from broken *Asclepias syriaca* (common milkweed) branches at Bernel Road Park, in Centre County, PA, USA in early to mid‐July 2023, and treatments of 0%, 2%, and 10% sap by volume were prepared in a 50% sugar solution using serial dilution. These concentrations were chosen based on preliminary assays to be below levels that would make bumble bees very sick or cause mortality. Milkweed sap has a concentration of ~1.3% cardenolides (w/w) (Zalucki et al. 2001), thus these assessed doses correspond approximately to 0%, 0.026%, and 0.13% cardenolide respectively.

#### 2.2.3 Milkweed Nectar

To better assess the impacts of field milkweed nectar, experiments were also run on nectar collected from floral heads of common milkweed plants ( *Asclepias syriaca* ). *A. syriaca* has been reported to contain about 1.4 ng/μL of cardenolides in its nectar (Villalona et al. 2020) and *Asclepias* in general averages at 14.7 ng/uL (Manson et al. 2012), thus this sample most likely was 0.0001%–0.0015% cardenolide. For this, nectar was collected at Bernel Road Park, in Centre County, PA in early‐ to mid‐July 2023. Multiple floral heads were covered in mesh bags overnight to prevent night feeding from moths or other nocturnal pollinators, and floral heads were removed in the morning and brought back to the lab, where individual flowers were inserted into a capsule upside down and centrifuged to extract the nectar. The pooled nectar had a sugar content of 20%. Given this lower sugar concentration compared to prior trials, and to enable comparison of results to known cardenolide concentrations, the results from this nectar were compared to four ouabain treatment solutions mixed at 20% sugar content: 0%, 0.00001%, 0.0001%, 0.01%. As milkweed nectar contains additional substances besides sucrose and cardenolide, we also chose two monofloral (according to producer) honeys as control solutions that were diluted to 20% sugar content, including buckwheat (Gunter's, sourced from Berryville, VA) and fireweed (Made in Washington, sourced from Bellevue, WA). Milkweed nectar experiments were run on 12 *B. impatiens* workers per treatment.

#### 2.2.4 Natural Nectars

To test how natural nectars of various types, which likely contain variable secondary compounds, cardenolides or otherwise, impact heat tolerance in bumble bees, we ran heat treatments on 16–17 *B. impatiens* worker treatment groups fed several different honey types collected by *Apis mellifera* . Five local honeys were obtained and mixed with equal parts warm distilled water to create a 50% sugar solution, which was confirmed using a refractometer. These included one multi‐floral spring blossom honey (Hickory, PA), and four non‐organic honeys marketed as largely monofloral (according to producer): buckwheat (Berryville, VA), knotweed (Hickory, PA) orange blossom (Victor, NY), and common milkweed (Linesville, PA). These honeys were chosen because they are widely different plant lineages and designated as monofloral, not based on known compounds in nectar. The common milkweed honey could represent another source of milkweed nectar; however, the degree to which this honey is exclusively milkweed honey was not confirmed. Honey is processed by honey bees via enzyme‐catalyzed and non‐enzymatic reactions, and chemical composition differs from nectars found in nature (Wang and Li 2011; du Rand et al. 2015); thus these may not represent compounds encountered by bumble bees upon original exposure. We assumed source by what was declared on the purchased honey and did not confirm fidelity to source.

#### 2.2.5 Ouabain in *B. griseocollis*

To test whether the milkweed specialist *B. griseocollis* would be less impacted by cardenolides, we ran heat trials on *B. griseocollis* from the two colonies, treated with three concentrations of ouabain: 0%, 0.01%, and 0.1%, and compared the results to a random subsampling (to enable equal sample sizes to *B. griseocollis* ) of *B. impatiens* at these doses. These concentrations were chosen for direct comparison to assess the control, the field‐realistic dose, and the highest dose for more pronounced effects. Twelve workers of each species were tested per treatment.

### 2.3 Feeding Procedure

Worker bees were randomly chosen (were not selected by age) and removed from the colonies with forceps and placed individually into separate cages. The cages were upside‐down round plastic containers (473 mL) with drilled air and access holes, and a small hole drilled into the bottom for provisioning nectar (diagram in Figure S1). Each caged bee was left undisturbed in the dark for a 2‐h starvation period, after which a single treatment of 950 μL of solution was administered, and the bees were allowed to feed on their treatment solution for 24 h. Solutions were fed from 1.5 mL upside‐down plastic snap‐top microcentrifuge tubes with a hole drilled in the bottom from which the bees could feed. Consumption was recorded by subtracting the vial weights post‐consumption from the weight of the vials with initial treatment added. For each trial, 12–17 bees were tested per treatment (see replicate numbers above), using 5–6 bees from each colony per concentration or treatment group. For the milkweed sap experiments, 5 bees per treatment were tested, with each treatment kept in a small cage together rather than in isolation and allowed to feed for 24 h, with no prior starvation period.

### 2.4 Thermal Trial Procedure

After feeding for 24 h, the bees were transferred to individual vials in a way that minimized disturbance and then were transferred to glass test tubes and inserted into one of two water baths (Benchmark, MyBath 4 L, model H2000‐4 and B2000‐4) (setup diagrammed in Figure S1). The test tubes were sealed at the top with foam plugs (Genesee Scientific, Morrisville, NC, USA), typically used for *Drosophila* research to allow for airflow, and the water bath was set to 43.8°C to achieve a confirmed critical maximum temperature of 43°C within the vial (Oyen and Dillon 2018). This was found to be the ideal temperature to capture a full range of responses over the course of 3 h (Feuerborn et al. 2023) as lower temperatures resulted in heat‐resistant bees (i.e., had not reached heat stupor after 3 h and thus were susceptible to confounding starvation effects). Each trial was run with all 15 tubes in place, with a thermometer inside one to measure temperature throughout the trial. Treatments and colonies were randomly split up across runs to account for run effects. Every 5 min, the water bath was opened, and the bees were checked individually for heat stupor, and time to heat stupor (THS), in minutes, was recorded. Heat stupor was identified by the inability to right itself, with twitching limbs and slow uncoordinated movements, indicating the loss of neuromuscular control (Martinet et al. 2015). Heat stupor was the chosen metric for thermal tolerance because the time that a bee first expresses heat stress can be subjective and difficult to pinpoint. Heat stupor can be consistently and reliably identified.

### 2.5 Mortality Tests

To test the impact of different ouabain concentrations on bumble bee health and activity levels, five concentrations of ouabain (0.0%, 0.0001%, 0.001%, 0.01%, 0.10%) were administered to 10 *B. impatiens* workers per treatment. Bees were fed individually and allowed to feed undisturbed for 24 h after a 2‐h starvation period using the same setup as the thermal trials, with the observation of activity occurring at the end of the feeding period. Activity levels were recorded and categorized in the following manner: alive (alert, mobile), lethargic (only moves in response to manual prodding), sick (no movement in response to stimulus, but able to lift a limb), dead (no movement or response).

### 2.6 Statistical Methods

All statistical analyses were conducted in R (version 4.2.1). All models met assumptions of residual normality (Shapiro–Wilk) and homogeneity of variance (Levene's). Model selection was performed using Akaike's Information Criterion corrected for small sample size (AICc) with the AICcmodavg package (Mazerolle 2017). The significance of the fixed effects was assessed via analysis of variance (ANOVA). Post hoc pairwise comparisons of ouabain concentration effects on the estimated marginal means (EMM) of THS were conducted using the emmeans package (Lenth et al. 2022), with Tukey's adjustment for multiple comparisons. To evaluate the effects of ouabain concentration on THS in *B. impatiens* , we fitted a linear mixed‐effects model (LMM) with ouabain concentration and nectar consumption as fixed effects (to account for their potential influence on THS), and colony as a random effect. An additive model was chosen (THS ~ ouabain concentration + nectar consumption + (1|Colony)), as it had a lower AICc score (ΔAICc > 2) than the interactive model (THS ~ ouabain concentration*nectar consumption + (1|Colony)). The same statistical methods were applied to the comparisons of ouabain, milkweed nectar, and honey at 20% sugar. For the *B. griseocollis* experiment, the analysis included a linear mixed‐effects model testing the interactive effects of bee species and ouabain concentration, the additive effect of consumption, and the random effect of colony. The models used for our reported data did not include a run effect as we balanced treatments within runs, confirmed the consistency of controls across runs, and analyses with run included in the model did not change patterns of significance.

To evaluate the effects of milkweed sap on THS in *B. impatiens* , we used a linear model testing the effect of sap concentration without including consumption as a factor, as the simpler model had the lowest AIC score in both cases, or colony as it did not explain any of the variance. To evaluate the effects of different honeys (50% sugar/sucrose) on THS, we first fit a linear regression model of THS as a function of the interaction between consumption and each honey type (consumption * honey type). As there was a marginally significant effect of the interaction term, we then explored the effect of consumption on THS for each honey type individually as well as all non‐milkweed honeys combined using estimated marginal trends (EMTs) from the *emmeans* package. For the honeys and milkweed sap the effect of colony as a random effect was excluded in the final analysis because it did not explain any of the variance when it was included in an initial analysis.

## 3 Results

### 3.1 Effect of Ouabain Concentration on THS (50% Sugar)— *B. impatiens*

**FIGURE 1:**
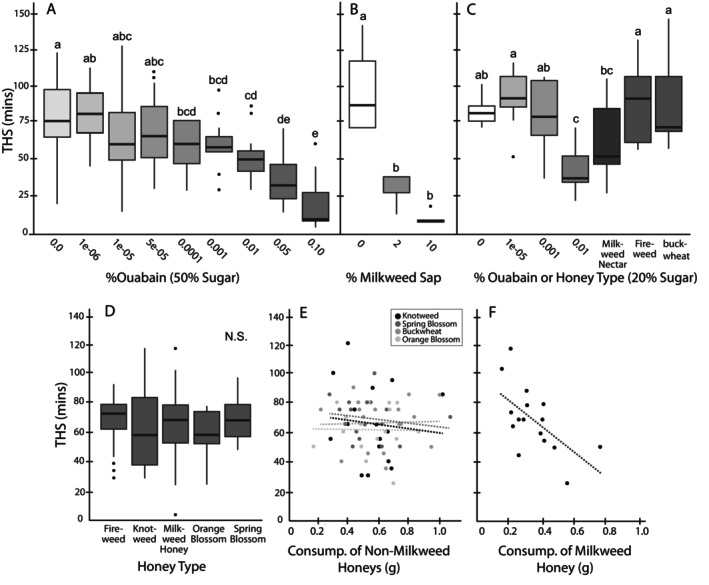
Differences in mean time to heat stupor (THS) in relation to cardenolide concentration and other treatments. This shows the effect of (A) ouabain concentrations in 50% sugar water on THS in bumble bees, (B) milkweed sap concentrations on THS in bumble bees, (C) ouabain concentrations in 20% sugar water, field‐collected milkweed nectar, and honey diluted to 20% sugar on THS in bumble bees, (D) different types of locally sourced honeys on THS in bumble bees, (E) consumption of each non‐milkweed honey on THS in bumble bees, and (F) consumption of milkweed honey on THS in bumble bees. Letters above box and whisker plots indicate statistically different groups (i.e., treatments with different letters have significant differences between them). Letters are comparable within but not across experiments.

We found a highly significant effect of ouabain concentration on THS in *B. impatiens* after controlling for consumption (*F*_8,152_ = 18.2, *p* < 0.001) (Figure 1A). Overall, there was a general decline in THS as ouabain concentration increased, with mean THS being the highest at 0.000001% ouabain (81.4 min ±5.18 [SE]) and lowest at 0.10% (17.5 min ±5.66). Concentrations of 0.00005% or lower did not yield significant differences from control (e.g., 0.00001% vs. control: *p* = 0.67), but concentrations of 0.0001% ouabain or higher yielded a significant decrease in THS compared to the control (e.g., 0.0001% vs. control: *p* = 0.024; 0.01% or greater: *p* < 0.001). There was no significant effect of consumption amount (*F*_1,148_ = 2.61, *p* = 0.11), although consumption showed a trend where it increased up to 0.001% and was the lowest at 0.10% (Figure S2). Statistical details for all analyses, including Estimated Marginal Means for THS and pairwise differences and significance values are presented in Table S1.

### 3.2 Effect of Milkweed Sap on THS— *B. impatiens*

The milkweed sap concentration had a significant effect on THS (*F*_2,12_ = 30.87, *p* < 0.001). Both 2% sap (36 min ±10.25) and 10% sap (17 min ±4.47) significantly reduced THS compared to the control group (106 min ±30.70) (*p* < 0.001 for both) (Figure 1B). However, THS did not differ significantly between the 2% and 10% treatments (95% CI [−50.83, 12.83], *p* = 0.29).

### 3.3 Effect of Milkweed Nectar and Natural Honeys on THS— *B. impatiens*

For the comparison of milkweed nectar with 20% honeys and ouabain concentrations, there was a significant effect of treatment type on estimated marginal mean (EMM) THS (*F*_6,72_ = 12.50, *p* < 0.001), but no effect of consumption amount (*F*_1,73_ = 0.32, *p* = 0.57). Bees that consumed field‐collected milkweed nectar (THS 56.8 min ±9.4) showed a trend towards lower heat tolerance than the control (THS 78.7 min ±9.4; *p* = 0.075), and significantly lower THS than the 0.0001% ouabain bees (*p* = 0.001; 89.6 min ±9.4) (Figure 1C). Milkweed nectar also significantly differed from the honey controls, with lower THS compared to fireweed (*p* = 0.006; 85.6 min ±9.4) and buckwheat (*p* = 0.003; 87.1 min ±9.4) and had a more similar THS to the field‐realistic ouabain dose (0.01%, *p* = 0.222).

In the 50% sugar multi‐honey assay, neither honey type (*F*_4,75_ = 0.55, *p* = 0.71) nor consumption (*F*_1,75_ = 0.85, *p* = 0.36) had a significant impact on mean THS, although there was a trend in their interaction (*F*_4,75_ = 2.05, *p* = 0.096) (Figure 1D). There was a negative correlation between the amount of consumption of milkweed honey and heat tolerance (Figure 1E,F): the more milkweed honey that was consumed, the lower the bee's heat tolerance (*p* = 0.005, *R* ^2^ = 0.39). Consumption did not affect THS when looking at all other honeys as a group (*p* = 0.89, *R* ^2^ = 0.0003), or individually (Buckwheat *p* = 0.89, *R* ^2^ = 0.001; Knotweed *p* = 0.58, *R* ^2^ = 0.010; Orange blossom *p* = 0.97, *R* ^2^ = 0.0001; Spring blossom *p* = 0.65, *R* ^2^ = 0.025).

### 3.4 Effect of Ouabain Concentration on THS in *B. griseocollis* and *B. impatiens* (50% Sugar)

**FIGURE 2:**
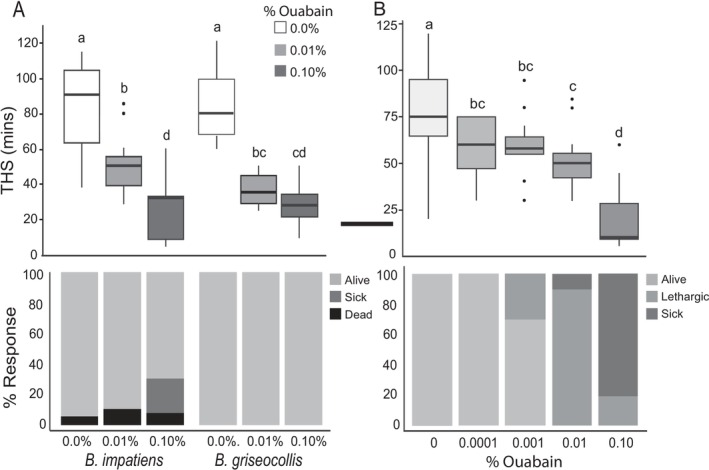
Comparison of effects on bees with time to heat stupor of different treatments. (A) Effect of ouabain concentrations on THS on *B. impatiens* and *B. griseocollis* and of ouabain concentrations on mortality in *B. impatiens* and *B. griseocollis* with mortality data as reported by Villalona et al. (2020). (B) Effect on time to heat stupor (THS) (top) and mortality (bottom) for ouabain using mortality obtained in the present study. Letters above box and whisker plots indicate statistically different groups (i.e., treatments with different letters have significant differences between them). Letters are comparable within but not across experiments.

We found a significant effect of concentration when comparing across both species (*F*_2,74_ = 81.89, *p* < 0.001), whereby all three concentrations were significantly different from each other (*p* < 0.001). There was no effect of species alone (*F*_1,28_, = 1.04, *p* = 0.32) and in the interactions, species did not differ in THS when concentration was the same. There was a significant interaction between species and concentration overall (*F*_1,73_ = 3.41, *p* = 0.038), as *B. griseocollis* at 0.01% and 0.10% ouabain concentration were not significantly different (Figure 2A). There was a trend towards an effect of consumption on THS (*F*_1,76_, = 3.35, *p* = 0.071), as both species tended to consume less of the 0.1% diet.

### 3.5 Ouabain Toxicity

*B. impatiens* workers were fully active when fed the control or 0.0001% ouabain treatment, while a minority of bees were lethargic when fed 0.001% ouabain, most were lethargic with the 0.01% treatment, and most were sick at 0.1% (Figure 2B). A reduction in heat tolerance occurred when bees were fully active (at 0.0001%), thus reduced heat tolerance does not align solely with obvious signs of illness. However, those that are fully lethargic (0.001%) or sick (0.1%) showed more drastic reductions in heat tolerance (Figure 2B), suggesting this level of illness corresponds to more pronounced effects on heat tolerance. Prior comparisons between *B. impatiens* and *B. griseocollis* that measured sickness but not lethargy found that *B. griseocollis* , unlike *B. impatiens* , was not sick at any of these concentrations (Villalona et al. 2020), yet our data show that ouabain concentrations had similar effects on heat tolerance in *B. griseocollis* and *B. impatiens* (Figure 2A).

## 4 Discussion

Climate change has been found to be a leading factor in bumble bee declines (Kerr et al. 2015; Soroye et al. 2020; Ghisbain et al. 2021; Martínez‐López et al. 2021; Ghisbain et al. 2023; Janousek et al. 2023). Some of the simplest solutions for alleviating the effects of global warming on bumble bees are altering the nutrition available to them in their landscapes; thus, understanding whether food resources impact thermal response is important for their management. Here, we sought to specifically address whether certain secondary compounds in nectar may impact heat tolerance, focusing on cardenolide toxins common in milkweeds (Apocynaceae) and several other plant families. We found that cardenolides have a significant impact on heat tolerance in bumble bees, even at concentrations lower than typical field‐realistic concentrations; thus bees living on diets with considerable milkweed nectar are likely to have reduced thermal tolerance. In contrast, bees fed different honeys did not differ in thermal tolerance; thus most nest‐fed honey likely has low impact on thermal tolerance in bees.

Our data showed that field‐realistic levels of milkweed cardenolides likely impact heat tolerance in these bees. Thus, even if bees mix milkweed nectar with other plants, they may be impacted. While it is possible that low doses of the toxin could have generated a stimulatory hormetic response, our data showed low concentrations of cardenolides still yielded a diminished THS and the lowest levels yielded no difference from controls, suggesting that if there are benefits, these would involve a very small effect not detectable with our sampling.

The majority of the tests performed in this study used ouabain, a commercially available cardenolide. To determine whether this cardenolide represents the toxicity of natural milkweed toxins, we conducted tests using milkweed sap, a known source of concentrated cardenolides. The milkweed sap had significant impacts on heat tolerance in *B. impatiens* , confirming that naturally occurring cardenolides and ouabain incur similar effects. The more dramatic effects of milkweed sap may be a result of higher concentrations of cardenolide exposure, as suggested by the much higher levels in sap in the literature (Züst et al. 2019), with the 10% solution being similar in concentration to our highest ouabain dose. Results from field‐collected milkweed nectar matched the THS values yielded from ouabain from a similar concentration thought to occur in milkweed nectars, confirming that ouabain is a reliable proxy for milkweed cardenolides and that field milkweed nectar has a negative effect on heat tolerance in these bees. Prior research, however, noted that *B. impatiens* experienced lower levels of toxicity from the predominant natural cardenolide in milkweed nectar when compared to ouabain (Jones et al. 2023).

We also sought to determine the degree to which nectar diets in the field in general might impact thermal tolerance, as perhaps other compounds may play a role in thermal tolerance. Generalist pollinators like bumble bees typically sample several flower species, with 1–9 species visited per foraging trip (Yourstone et al. 2023), thus impacts of nectar on heat tolerance will depend on the degree to which the community of floral nectars impacts heat response. We found that honey type had no significant impact on heat tolerance in *B. impatiens*. Our diverse “spring” diet had the same effect as monofloral honeys, which may indicate that most floral compounds do not impact heat tolerance. However, monofloral diets are unlikely to be from a single source which may dilute any effects of toxic flowers. These results suggest secondary compounds in nectar, at least from processed honey stores, are unlikely to play a major role in the heat tolerance of generalist bees.

The honey labeled as Common Milkweed honey did not show the same effect as our field‐collected milkweed nectar when diluted to the same concentration. We noticed a trend, however, where only milkweed honey resulted in reduced THS with increased consumption, suggesting there is a low level of cardenolide in the honey. Honey bees are known to filter out certain compounds, particularly metals, during the production of honey (du Rand et al. 2015; Borsuk et al. 2021), thus milkweed honeys and other honeys may already have had some of their secondary compounds removed. The milkweed honey may also have been diluted by visits to more diverse nectar sources than milkweed. Nevertheless, this suggests that diets that skew more towards milkweed plants will decrease the ability to thermoregulate in a heat stress event.

The relationship between cardenolide exposure and heat tolerance may have greater implications for species that favor milkweed, such as *B. griseocollis*, which consume more milkweed nectar than nectar from other plants, with about 87% of their plants visited found to be milkweed (Villalona et al. 2020). We theorized that due to their specialized relationship, these bees may be better able to metabolize the toxins to which they are being exposed. However, *B. griseocollis* ' thermal tolerance was as impacted by field levels of toxins as *B. impatiens* , despite *B. griseocollis* exhibiting lower levels of sickness or lethargy after consumption (Villalona et al. 2020). *B. griseocollis* in the Eastern U.S. has been found to be among the most heat‐tolerant bumble bee species in lab assays (Martinet et al. 2020; Feuerborn et al. 2023). It is possible that the increased natural heat tolerance in this bee can counter the interaction of heat stress and cardenolide exposure. Both *B. impatiens* and *B. griseocollis* have exceptionally high natural thermal tolerance (Feuerborn et al. 2023) and are not species considered to be vulnerable (Hatfield et al. 2014; Hatfield et al. 2015) so it would be interesting to see the role such compounds might play in more heat‐susceptible species. Data from this work could also be compared to other milkweed specialists, such as monarchs, to understand how their heat response may relate to cardenolide exposure.

One might suspect that any illness reduces THS, and that the effect we see with cardenolides could be a response to illness, given that reduced motor abilities played a role in measuring both sickness and heat tolerance. With cardenolides, we found significant heat responses with doses 10 times less than those that induce lethargy, but we did find that doses inducing lethargy and sickness caused increasingly dramatic effects on heat tolerance. Cardenolides impact neural and heart function by inhibiting the Na^+^/K^+^‐ATPase pump, disrupting the ion gradient across the cell membrane (Dobler et al. 2011; Petschenka et al. 2018). This effect on the heart may have led to a faster decline of motor abilities and internal effects not visible with behavior at lower doses. Even if motor abilities explain this pattern, the results have important implications: bees that ingest cardenolides in the field may exhibit reduced activity at elevated temperatures compared to bees that have not. Further research is needed to understand the mechanisms behind these processes. In particular, it would be helpful to learn which heat shock proteins are being activated or inhibited during a heat stress response under the influence of these toxins and thus how nutrition generates this physiological effect.

Ultimately, these findings suggest that while naturally occurring cardenolides found in floral nectars do inhibit a bee's ability to thermoregulate in heat stress events, most generalist bees likely obtain a diet that is diverse enough to counter any effects of nectar compounds. In contrast, specialist species on cardenolide‐rich plants may be more vulnerable to foraging in heat. This study also highlights how certain compounds in nectars consumed by a bee may impact their individual response to heat waves and could explain some of the variance found in heat tolerance in field‐collected bees.

## Author Contributions

**Rachael Shippee:** conceptualization (equal), data curation (lead), formal analysis (equal), investigation (lead), visualization (lead), writing – original draft (lead), writing – review and editing (supporting). **Cody Feuerborn:** conceptualization (supporting), data curation (supporting), formal analysis (equal), visualization (supporting), writing – original draft (supporting), writing – review and editing (supporting). **Allie Bradley:** conceptualization (supporting), data curation (supporting), investigation (supporting), writing – original draft (supporting), writing – review and editing (supporting). **Hannah Jahnke:** conceptualization (supporting), data curation (supporting), investigation (supporting), writing – original draft (supporting), writing – review and editing (supporting). **Heather M. Hines:** conceptualization (equal), funding acquisition (lead), project administration (lead), visualization (supporting), writing – original draft (supporting), writing – review and editing (lead).

## Conflicts of Interest

The authors declare no conflicts of interest.

## Supporting information


**Data S1:** ece372420‐sup‐0001‐Supinfo01.xlsx.

**Table S1:** ece372420‐sup‐0002‐TableS1.docx.

**Figure S1:** ece372420‐sup‐0003‐FigureS1.docx

**Figure S2:** ece372420‐sup‐0004‐FigureS2.docx

## Data Availability

All data including THS data and activity metrics is available in the Data S1.
