# Supplementary material for: Secondary Compounds in Milkweed Nectar Negatively Impact Thermal Tolerance in Bumble Bees
Source: Ecol Evol. 2025 Nov 9;15(11):e72420. doi: 10.1002/ece3.72420 (PMC12597253; doi:10.1002/ece3.72420)
Supplement: Supplementary file 2 — Table S1: ece372420‐sup‐0002‐TableS1.docx. [file ECE3-15-e72420-s004.docx]

**Table S1.** Statistics for Estimated Marginal Means (EMM) for THS and pairwise differences*.* Significance code: 0 *** 0.001 ** 0.01 * 0.05 . (marginal significance)

(A) *B. impatiens* Ouabain (50% sugar) Estimated Marginal Means (EMM).

| Concentration | EMM | SE | df | 95% CI |
| --- | --- | --- | --- | --- |
| 0.00% | 79.69 | 3.95 | 24.49 | [71.55, 87.83] |
| 0.000001% | 81.43 | 5.18 | 60.05 | [71.06, 91.80] |
| 0.00005% | 66.37 | 5.16 | 61.71 | [56.05, 76.68] |
| 0.00001% | 69.82 | 4.93 | 49.78 | [59.92, 79.73] |
| 0.0001% | 58.92 | 5.80 | 59.90 | [47.31, 70.52] |
| 0.001% | 57.18 | 5.62 | 52.48 | [45.91, 68.44] |
| 0.01% | 51.81 | 5.79 | 59.81 | [40.23, 63.39] |
| 0.05% | 34.82 | 5.61 | 59.14 | [23.60, 46.04] |
| 0.10% | 17.53 | 5.66 | 61.77 | [6.22, 28.84] |

(B) *B. impatiens* Ouabain (50% sugar) pairwise differences.

| Comparison | estimate | SE | df | t-ratio | p value |
| --- | --- | --- | --- | --- | --- |
| 0.00% - 0.000001% | -1.74 | 5.71 | 161.80 | -0.30 | 1.000 |
| 0.00% - 0.00005% | 13.32 | 5.69 | 160.83 | 2.34 | 0.323 |
| 0.00% - 0.00001% | 9.87 | 5.42 | 161.32 | 1.82 | 0.669 |
| 0.00% - 0.0001% | 20.77 | 6.12 | 162.52 | 3.39 | **0.024 *** |
| 0.00% - 0.001% | 22.51 | 5.90 | 162.54 | 3.82 | **0.006 **** |
| 0.00% - 0.01% | 27.88 | 6.11 | 162.54 | 4.56 | **<0.001 ***** |
| 0.00% - 0.05% | 44.87 | 6.01 | 162.48 | 7.46 | **<0.001 ***** |
| 0.00% - 0.10% | 62.16 | 6.09 | 162.64 | 10.21 | **<0.001 ***** |
| 0.000001% - 0.00005% | 15.06 | 6.52 | 157.88 | 2.31 | 0.341 |
| 0.000001% - 0.00001% | 11.61 | 6.30 | 157.46 | 1.84 | 0.655 |
| 0.000001% - 0.0001% | 22.51 | 7.26 | 159.56 | 3.10 | 0.056 |
| 0.000001% - 0.001% | 24.25 | 7.08 | 159.31 | 3.43 | **0.022 *** |
| 0.000001% - 0.01% | 29.62 | 7.26 | 159.33 | 4.08 | **0.002 **** |
| 0.000001% - 0.05% | 46.61 | 7.12 | 159.72 | 6.55 | **<0.001 ***** |
| 0.000001% - 0.10% | 63.90 | 7.16 | 159.63 | 8.92 | **<0.001 ***** |
| 0.00005% - 0.00001% | -3.46 | 6.31 | 157.30 | -0.55 | 1.000 |
| 0.00005% - 0.0001% | 7.45 | 7.22 | 161.06 | 1.03 | 0.982 |
| 0.00005% - 0.001% | 9.19 | 7.04 | 161.08 | 1.31 | 0.929 |
| 0.00005% - 0.01% | 14.56 | 7.21 | 161.04 | 2.02 | 0.532 |
| 0.00005% - 0.05% | 31.55 | 7.05 | 161.25 | 4.47 | **<0.001 ***** |
| 0.00005% - 0.10% | 48.84 | 7.09 | 161.14 | 6.89 | **<0.001 ***** |
| 0.00001% - 0.0001% | 10.91 | 6.98 | 159.78 | 1.56 | 0.823 |
| 0.00001% - 0.001% | 12.65 | 6.73 | 160.03 | 1.88 | 0.629 |
| 0.00001% - 0.01% | 18.02 | 6.98 | 159.73 | 2.58 | 0.203 |
| 0.00001% - 0.05% | 35.00 | 6.95 | 159.83 | 5.04 | **<0.001 ***** |
| 0.00001% - 0.10% | 52.29 | 7.05 | 159.62 | 7.42 | p<0.001 |
| 0.0001% - 0.001% | 1.74 | 6.92 | 157.07 | 0.25 | 1.000 |
| 0.0001% - 0.01% | 7.11 | 7.11 | 156.62 | 1.00 | 0.985 |
| 0.0001% - 0.05% | 24.10 | 7.09 | 157.19 | 3.40 | **0.024 *** |
| 0.0001% - 0.10% | 41.39 | 7.18 | 157.84 | 5.76 | **<0.001 ***** |
| 0.001% - 0.01% | 5.37 | 6.92 | 157.01 | 0.78 | 0.997 |
| 0.001% - 0.05% | 22.36 | 6.98 | 158.49 | 3.20 | **0.042 *** |
| 0.001% - 0.10% | 39.65 | 7.10 | 159.40 | 5.58 | **<0.001 ***** |
| 0.01% - 0.05% | 16.99 | 7.07 | 157.17 | 2.40 | 0.291 |
| 0.01% - 0.10% | 34.28 | 7.16 | 157.82 | 4.79 | **<0.001 ***** |
| 0.05% - 0.10% | 17.29 | 6.89 | 156.64 | 2.51 | 0.236 |

(C) *B. impatiens* milkweed nectar, honeys, ouabain, at 20% sugar Estimated Marginal Means (EMM).

| Conc. or Type | EMM | SE | df | 95% CI |
| --- | --- | --- | --- | --- |
| milkweed | 56.75 | 9.41 | 3.75 | [29.94, 83.56] |
| fireweed | 85.61 | 9.43 | 3.76 | [58.77, 112.44] |
| buckwheat | 87.05 | 9.40 | 3.73 | [60.20, 113.91] |
| 0.00% | 78.67 | 9.43 | 3.76 | [51.83, 105.50] |
| 0.00001% | 89.57 | 9.40 | 3.73 | [62.71, 116.43] |
| 0.0001% | 77.34 | 9.74 | 4.27 | [50.96, 103.71] |
| 0.01% | 38.45 | 9.46 | 3.81 | [11.66, 65.24] |

(D) *B. impatiens* milkweed nectar, honeys, ouabain, at 20% sugar pairwise differences.

| Comparison | estimate | SE | df | t-ratio | p value |
| --- | --- | --- | --- | --- | --- |
| milkweed - fireweed | -28.86 | 7.62 | 72.06 | -3.79 | **p=0.006 **** |
| milkweed - buckwheat | -30.30 | 7.55 | 72.01 | -4.01 | **p=0.003 **** |
| milkweed - 0.00% | -21.92 | 7.62 | 72.04 | -2.87 | 0.075 |
| milkweed - 0.00001% | -32.82 | 7.55 | 72.01 | -4.34 | **p=0.001 ***** |
| milkweed - 0.0001% | -20.59 | 7.93 | 72.07 | -2.60 | 0.142 |
| milkweed - 0.01% | 18.30 | 7.69 | 72.06 | 2.38 | 0.222 |
| fireweed - buckwheat | -1.45 | 7.56 | 72.03 | -0.19 | 1.000 |
| fireweed - 0.00% | 6.94 | 7.54 | 72.02 | 0.92 | 0.968 |
| fireweed - 0.00001% | -3.96 | 7.56 | 72.03 | -0.52 | 0.998 |
| fireweed - 0.0001% | 8.27 | 7.94 | 72.02 | 1.04 | 0.942 |
| fireweed - 0.01% | 47.16 | 7.56 | 72.02 | 6.24 | **p<0.001 ***** |
| buckwheat - 0.00% | 8.39 | 7.56 | 72.01 | 1.11 | 0.923 |
| buckwheat - 0.00001% | -2.52 | 7.53 | 72.00 | -0.33 | 1.000 |
| buckwheat - 0.0001% | 9.72 | 7.91 | 72.03 | 1.23 | 0.881 |
| buckwheat - 0.01% | 48.60 | 7.60 | 72.03 | 6.40 | **p<0.001 ***** |
| 0.00% - 0.00001% | -10.90 | 7.56 | 72.01 | -1.44 | 0.777 |
| 0.00% - 0.0001% | 1.33 | 7.95 | 72.01 | 0.17 | 1.000 |
| 0.00% - 0.01% | 40.22 | 7.54 | 72.01 | 5.33 | **p<0.001 ***** |
| 0.00001% - 0.0001% | 12.23 | 7.91 | 72.03 | 1.55 | 0.717 |
| 0.00001% - 0.01% | 51.12 | 7.60 | 72.02 | 6.73 | **p<0.001 ***** |
| 0.0001% - 0.01% | 38.89 | 7.99 | 72.00 | 4.87 | **p<0.001 **** |

(E) *B. impatiens* honeys (50% sugar) Estimated Marginal Means (EMM).

| Type | EMM | SE | df | 95% CI |
| --- | --- | --- | --- | --- |
| Milkweed | 69.13 | 4.83 | 79 | [59.51, 78.75] |
| Buckwheat | 66.91 | 4.90 | 79 | [57.16, 76.66] |
| Orange Blossom | 61.51 | 4.82 | 79 | [51.91, 71.11] |
| Spring Blossom | 69.19 | 4.83 | 79 | [59.57, 78.80] |
| Knotweed | 63.27 | 4.83 | 79 | [53.65, 72.89] |

(F) *B. impatiens* honeys (50% sugar) pairwise differences.

| Comparison | estimate | SE | df | t.ratio | p.value |
| --- | --- | --- | --- | --- | --- |
| Milkweed - Buckwheat | 2.22 | 6.92 | 79 | 0.32 | 1.00 |
| Milkweed - Orange Blossom | 7.62 | 6.83 | 79 | 1.12 | 0.80 |
| Milkweed - Spring Blossom | -0.06 | 6.82 | 79 | -0.01 | 1.00 |
| Milkweed - Knotweed | 5.86 | 6.82 | 79 | 0.86 | 0.91 |
| Buckwheat - Orange Blossom | 5.40 | 6.87 | 79 | 0.79 | 0.93 |
| Buckwheat - Spring Blossom | -2.28 | 6.91 | 79 | -0.33 | 1.00 |
| Buckwheat - Knotweed | 3.64 | 6.92 | 79 | 0.53 | 0.98 |
| Orange Blossom - Spring Blossom | -7.68 | 6.83 | 79 | -1.12 | 0.79 |
| Orange Blossom - Knotweed | -1.76 | 6.83 | 79 | -0.26 | 1.00 |
| Spring Blossom - Knotweed | 5.92 | 6.82 | 79 | 0.87 | 0.91 |

(G) *B. impatiens* honeys (50% sugar) – Regression summary table comparing the effect of consumption on THS for each honey type and when grouping all other honeys (grouped Honeys) vs milkweed honey. Grouped Honeys include buckwheat, orange blossom, spring blossom, knotweed.

| Honey Type | Consumption trend | SE | df | t.ratio | p.value | R2 |
| --- | --- | --- | --- | --- | --- | --- |
| Milkweed | -96.0 | 33.0 | 75 | -2.91 | **p=0.005** | 0.3897 |
| Buckwheat | 2.9 | 22.8 | 75 | 0.13 | 0.90 | 0.0013 |
| Orange Blossom | -1.0 | 27.3 | 75 | -0.04 | 0.97 | 0.0001 |
| Spring Blossom | -11.8 | 26.1 | 75 | -0.45 | 0.65 | 0.0249 |
| Knotweed | 12.6 | 22.8 | 75 | 0.55 | 0.58 | 0.0097 |
| Grouped Honeys | 1.67 | 11.8 | 66 | 0.142 | 0.89 | 0.0003 |

(H) *B. impatiens* vs *B. griseocollis* ouabain (50% sugar) Estimated Marginal Means (EMM).

| Type | EMM | SE | df | 95% CI |
| --- | --- | --- | --- | --- |
| 0.00% | 88.34 | 4.6 | 9.29 | [77.98, 98.70] |
| 0.01% | 48.14 | 4.44 | 8.44 | [38.00, 58.28] |
| 0.10% | 22.76 | 4.54 | 10.27 | [12.69, 32.83] |
| griseocollis | 55.51 | 4.95 | 6.12 | [43.46, 67.56] |
| impatiens | 50.65 | 3.81 | 4.65 | [40.63, 60.67] |
| gris:0% | 92.99 | 6.68 | 18.69 | [79.00, 106.99] |
| gris:0.01% | 44.07 | 6.34 | 16.87 | [30.68, 57.47] |
| gris:0.1% | 29.47 | 6.23 | 18.74 | [16.41, 42.52] |
| imp:0% | 83.68 | 5.07 | 14.28 | [72.83, 94.53] |
| imp:0.01% | 52.21 | 5.13 | 15.12 | [41.28, 63.13] |
| imp:0.1% | 16.06 | 5.38 | 16.94 | [4.72, 27.41] |

(I) *B. impatiens* vs *B. griseocollis* ouabain (50% sugar) pairwise comparisons.

| Comparison | estimate | SE | df | t.ratio | p.value |
| --- | --- | --- | --- | --- | --- |
| gris - imp | 4.01 | 5.38 | 35.76 | 0.74 | 0.46 |
| 0.00% - 0.01% | 40.12 | 4.43 | 84.22 | 9.06 | **p<0.001***** |
| 0.00% - 0.10% | 65.25 | 5.39 | 87.27 | 12.11 | **p<0.001***** |
| 0.01% - 0.10% | 25.13 | 5.03 | 86.04 | 5.0 | **p<0.001***** |
| gris 0.00% - imp 0.10% | 75.75 | 8.56 | 79.50 | 8.85 | **p<0.001***** |
| imp 0.00% - imp 0.10% | 67.35 | 6.29 | 85.70 | 10.70 | **p<0.001***** |
| gris 0.00% - gris 0.10% | 63.16 | 7.52 | 86.23 | 8.40 | **p<0.001***** |
| gris 0.00% - gris 0.01% | 48.82 | 6.64 | 84.02 | 7.35 | **p<0.001***** |
| imp 0.00% - gris 0.10% | 54.75 | 7.47 | 84.56 | 7.33 | **p<0.001***** |
| imp 0.01% - imp 0.10% | 35.92 | 6.23 | 85.00 | 5.76 | **p<0.001***** |
| imp 0.00% - gris 0.01% | 40.41 | 7.29 | 78.59 | 5.55 | **p<0.001***** |
| imp 0.00% - imp 0.01% | 31.43 | 5.79 | 84.04 | 5.43 | **p<0.001***** |
| gris 0.00% - imp 0.01% | 39.84 | 7.61 | 78.98 | 5.23 | **p<0.001***** |
| gris 0.01% - imp 0.10% | 26.94 | 8.06 | 78.25 | 3.34 | **p=0.016*** |
| imp 0.01% - gris 0.10% | 23.32 | 7.42 | 85.25 | 3.14 | **p=0.027*** |
| gris 0.01% - gris 0.10% | 14.34 | 7.07 | 85.24 | 2.03 | 0.336 |
| gris 0.10% - imp 0.10% | 12.60 | 7.30 | 78.50 | 1.73 | 0.519 |
| gris 0.01% - imp 0.01% | -8.98 | 7.38 | 79.79 | -1.22 | 0.827 |
| gris 0.00% - imp 0.00% | 8.41 | 7.48 | 77.64 | 1.12 | 0.870 |
