# Supplementary material for: Secondary Compounds in Milkweed Nectar Negatively Impact Thermal Tolerance in Bumble Bees
Source: Ecol Evol. 2025 Nov 9;15(11):e72420. doi: 10.1002/ece3.72420 (PMC12597253; doi:10.1002/ece3.72420)
Supplement: Supplementary file 3 — Figure S1: ece372420‐sup‐0003‐FigureS1.docx [file ECE3-15-e72420-s002.docx]

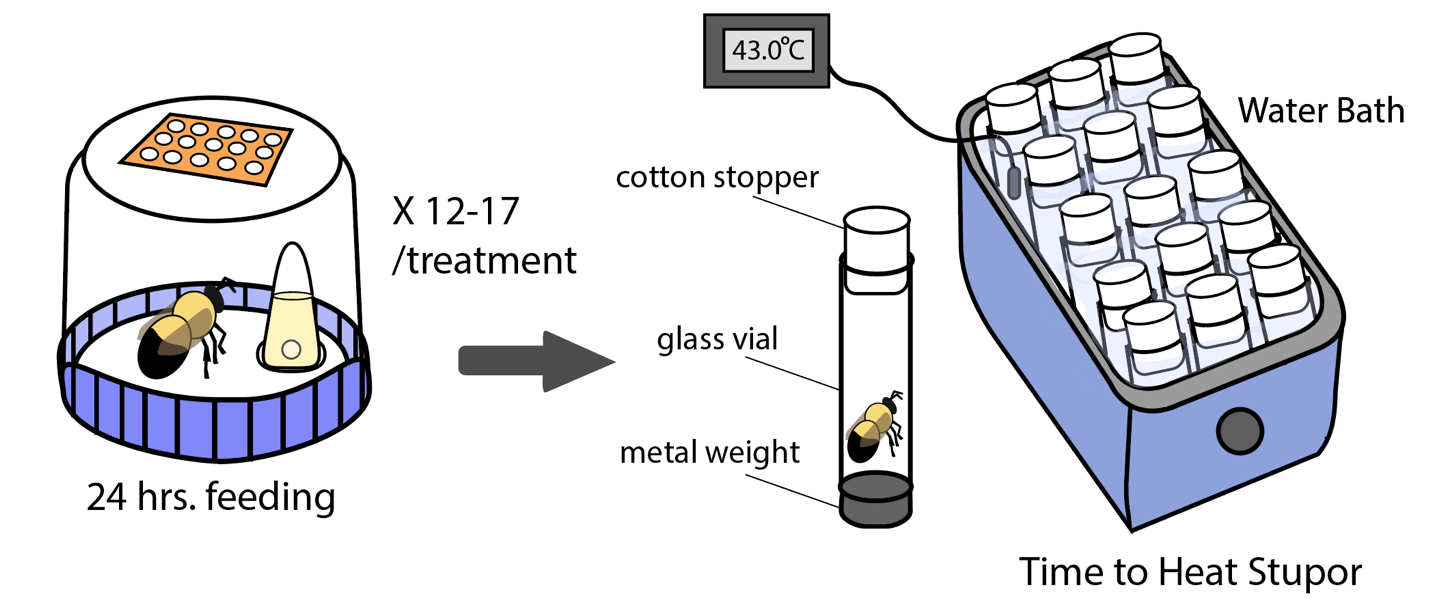


**Figure S1.** Schematic showing the setup for the feeding experiments and the setup for the time to heat stupor experiments (water bath had a lid to maintain heat and humidity). Feeding treatments are featured in Table 1.
