# Supplementary material for: Secondary Compounds in Milkweed Nectar Negatively Impact Thermal Tolerance in Bumble Bees
Source: Ecol Evol. 2025 Nov 9;15(11):e72420. doi: 10.1002/ece3.72420 (PMC12597253; doi:10.1002/ece3.72420)
Supplement: Supplementary file 4 — Figure S2: ece372420‐sup‐0004‐FigureS2.docx [file ECE3-15-e72420-s001.docx]

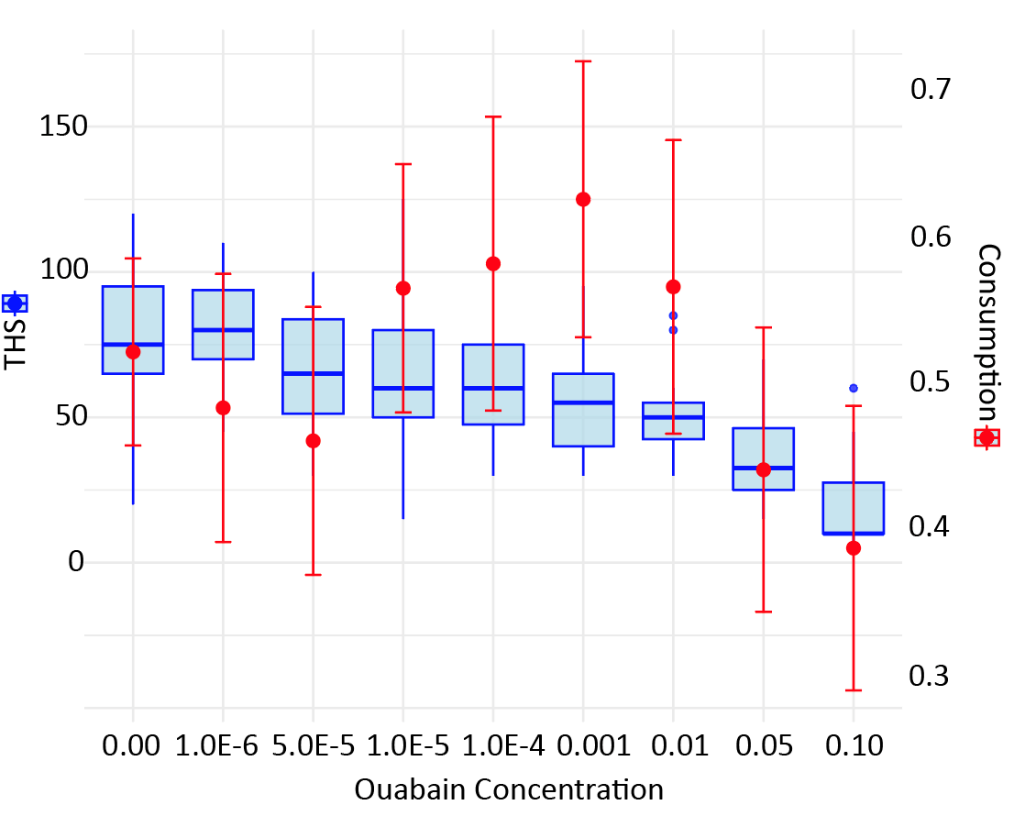


**Figure S2.** Comparison of consumption levels to THS response of the tested ouabain concentrations.
